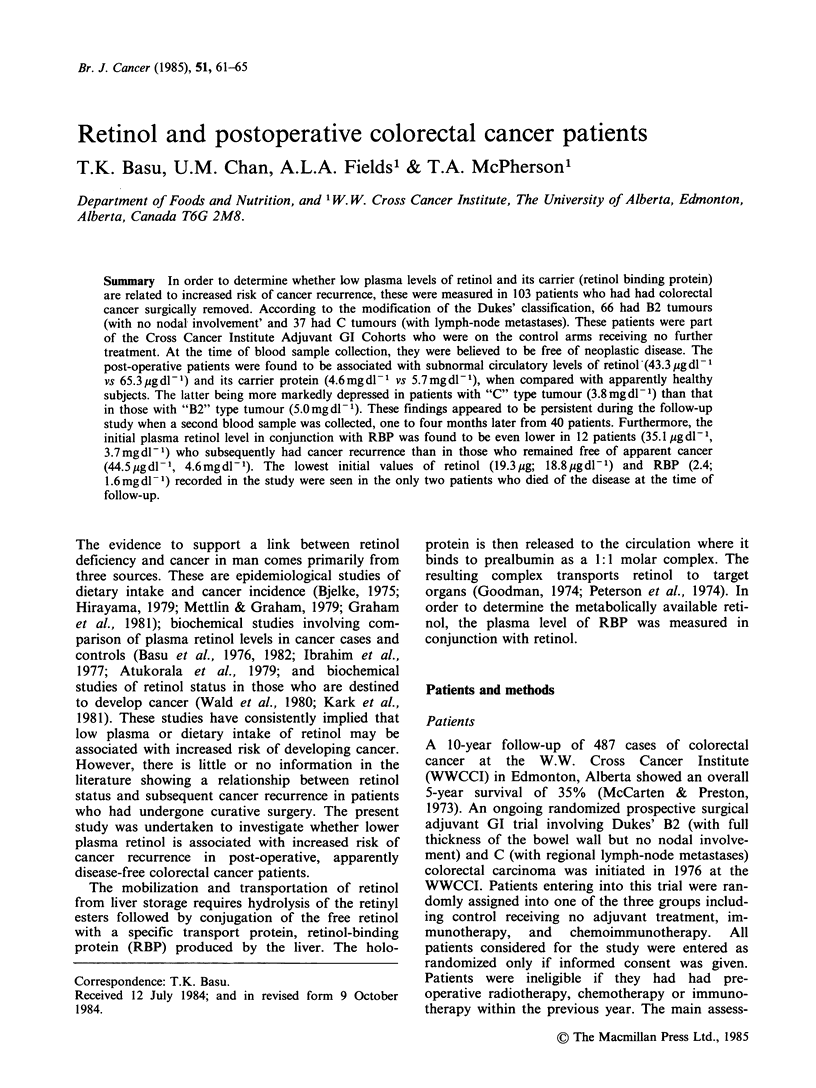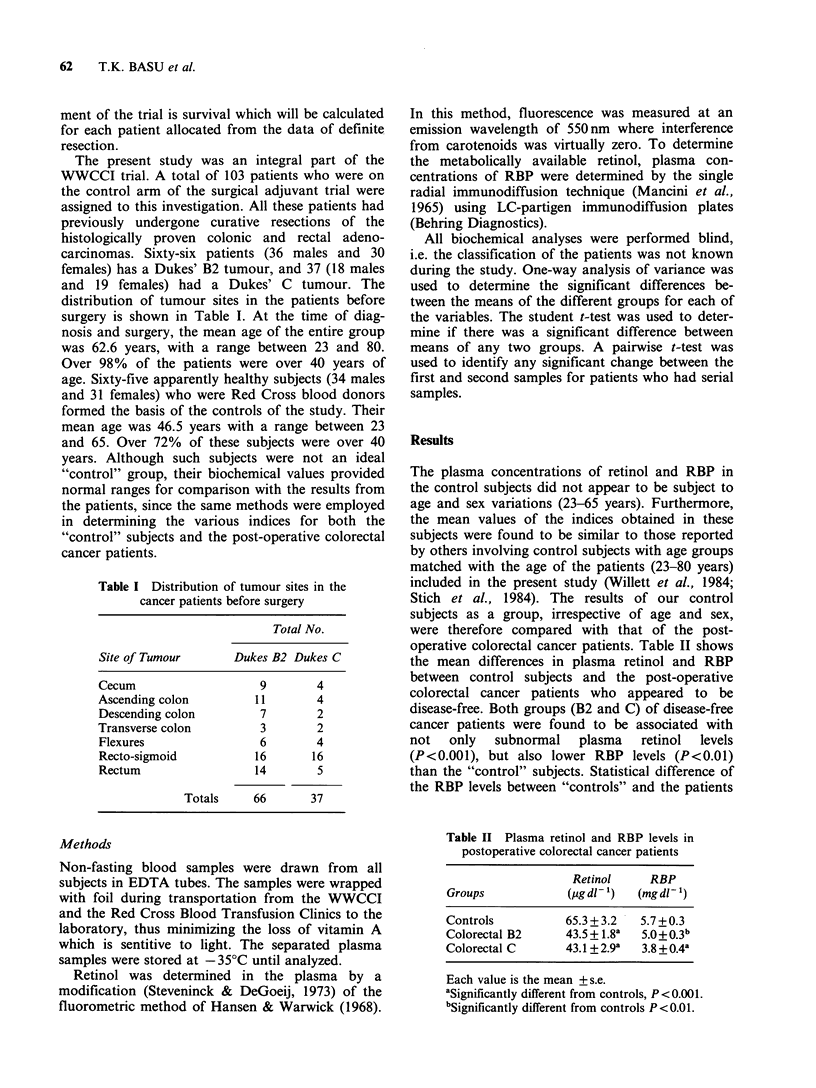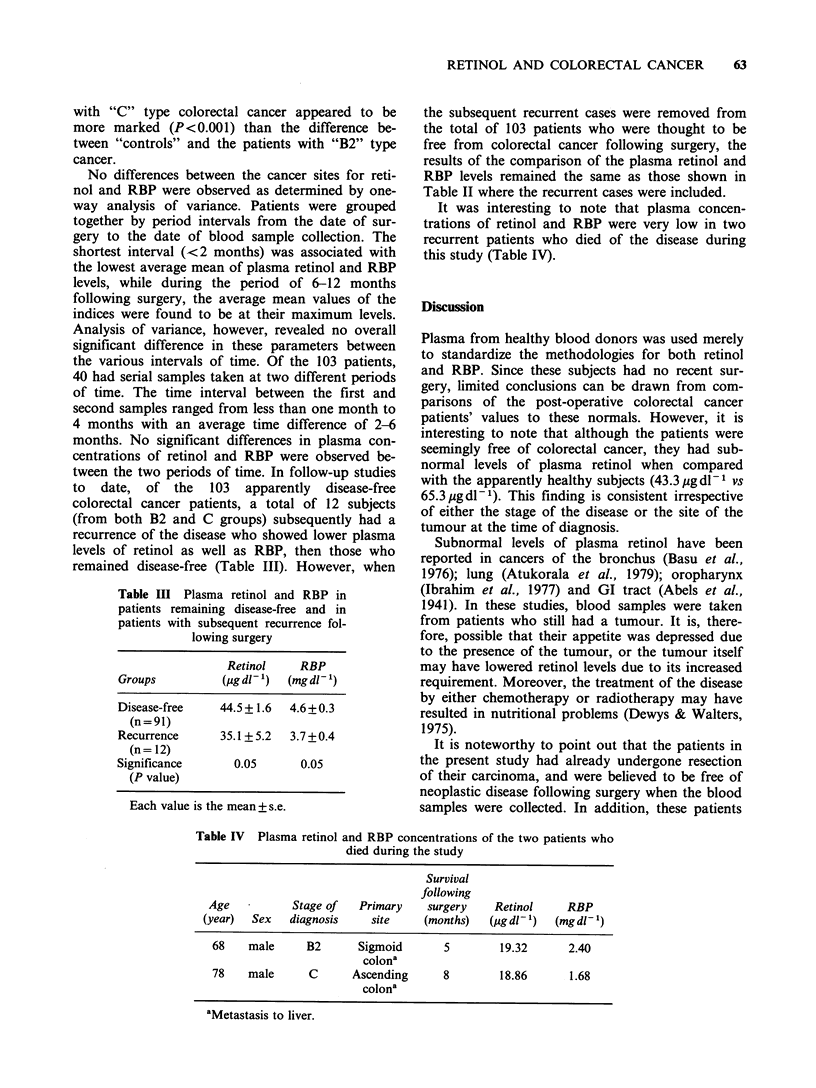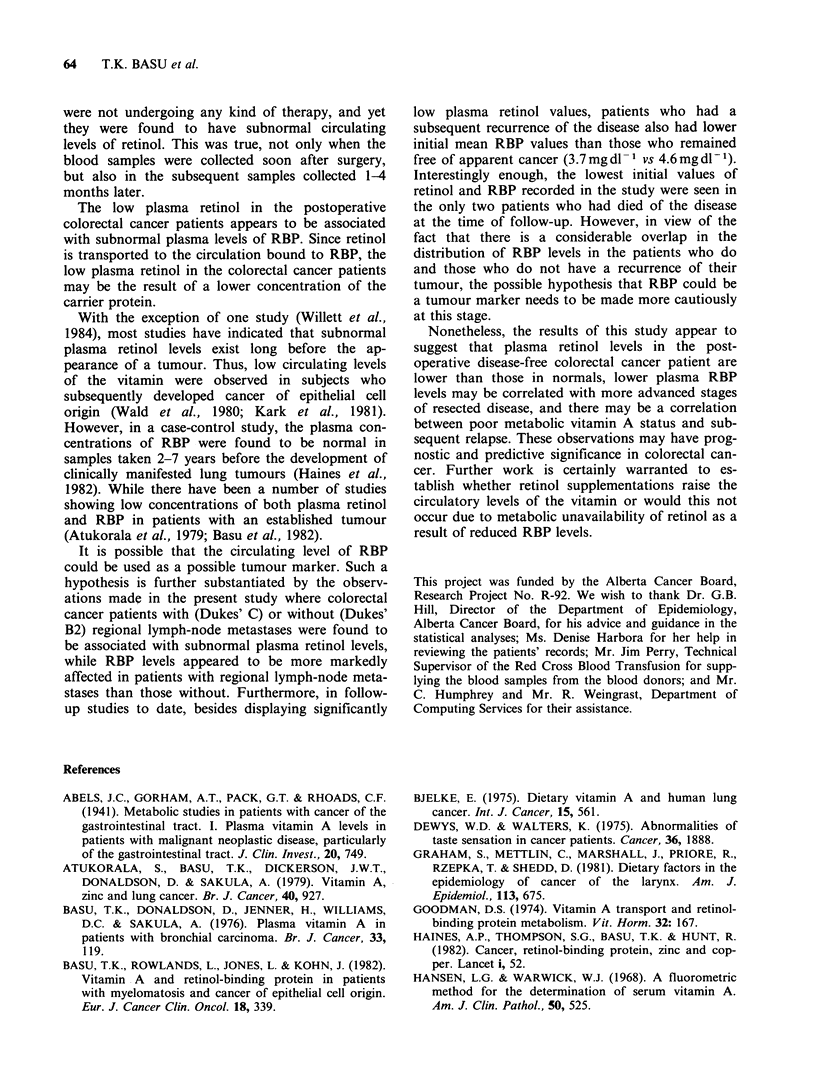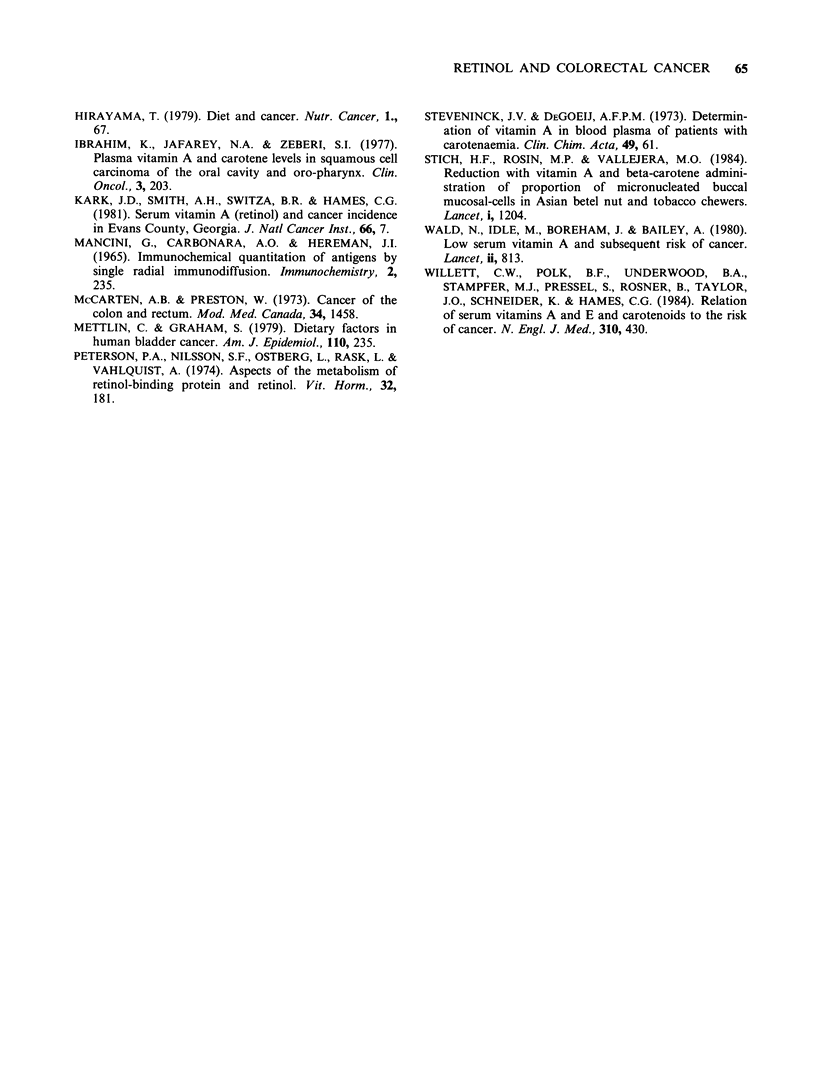# Retinol and postoperative colorectal cancer patients.

**DOI:** 10.1038/bjc.1985.9

**Published:** 1985-01

**Authors:** T. K. Basu, U. M. Chan, A. L. Fields, T. A. McPherson

## Abstract

In order to determine whether low plasma levels of retinol and its carrier (retinol binding protein) are related to increased risk of cancer recurrence, these were measured in 103 patients who had had colorectal cancer surgically removed. According to the modification of the Dukes' classification, 66 had B2 tumours (with no nodal involvement' and 37 had C tumours (with lymph-node metastases). These patients were part of the Cross Cancer Institute Adjuvant GI Cohorts who were on the control arms receiving no further treatment. At the time of blood sample collection, they were believed to be free of neoplastic disease. The post-operative patients were found to be associated with subnormal circulatory levels of retinol (43.3 micrograms dl-1 vs 65.3 micrograms dl-1) and its carrier protein (4.6 mg dl-1 vs 5.7 mg dl-1), when compared with apparently healthy subjects. The latter being more markedly depressed in patients with "C" type tumour (3.8 mg dl-1) than that in those with "B2" type tumour (5.0 mg dl-1). These findings appeared to be persistent during the follow-up study when a second blood sample was collected, one to four months later from 40 patients. Furthermore, the initial plasma retinol level in conjunction with RBP was found to be even lower in 12 patients (35.1 micrograms dl-1, 3.7 mg dl-1) who subsequently had cancer recurrence than in those who remained free of apparent cancer (44.5 micrograms dl-1, 4.6 mg dl-1). The lowest initial values of retinol (19.3 micrograms; 18.8 micrograms dl-1) and RBP (2.4; 1.6 mg dl-1) recorded in the study were seen in the only two patients who died of the disease at the time of follow-up.


*Br. J. Cancer* (1985), **51**, 61–65

### Retinol and postoperative colorectal cancer patients

T.K. Basu, U.M. Chan, A.L.A. Fields[1] & T.A. McPherson[1]

*Department of Foods and Nutrition, and [1]W.W. Cross Cancer Institute, The University of Alberta, Edmonton, Alberta, Canada T6G 2M8.*

**Summary** In order to determine whether low plasma levels of retinol and its carrier (retinol binding protein) are related to increased risk of cancer recurrence, these were measured in 103 patients who had had colorectal cancer surgically removed. According to the modification of the Dukes' classification, 66 had B2 tumours (with no nodal involvement' and 37 had C tumours (with lymph-node metastases). These patients were part of the Cross Cancer Institute Adjuvant GI Cohorts who were on the control arms receiving no further treatment. At the time of blood sample collection, they were believed to be free of neoplastic disease. The post-operative patients were found to be associated with subnormal circulatory levels of retinol (43.3 $\mu g\,dl^{-1}$ *vs* 65.3 $\mu g\,dl^{-1}$) and its carrier protein (4.6 $mg\,dl^{-1}$ *vs* 5.7 $mg\,dl^{-1}$), when compared with apparently healthy subjects. The latter being more markedly depressed in patients with "C" type tumour (3.8 $mg\,dl^{-1}$) than that in those with "B2" type tumour (5.0 $mg\,dl^{-1}$). These findings appeared to be persistent during the follow-up study when a second blood sample was collected, one to four months later from 40 patients. Furthermore, the initial plasma retinol level in conjunction with RBP was found to be even lower in 12 patients (35.1 $\mu g\,dl^{-1}$, 3.7 $mg\,dl^{-1}$) who subsequently had cancer recurrence than in those who remained free of apparent cancer (44.5 $\mu g\,dl^{-1}$, 4.6 $mg\,dl^{-1}$). The lowest initial values of retinol (19.3 $\mu g$; 18.8 $\mu g\,dl^{-1}$) and RBP (2.4; 1.6 $mg\,dl^{-1}$) recorded in the study were seen in the only two patients who died of the disease at the time of follow-up.

The evidence to support a link between retinol deficiency and cancer in man comes primarily from three sources. These are epidemiological studies of dietary intake and cancer incidence (Bjelke, 1975; Hirayama, 1979; Mettlin & Graham, 1979; Graham *et al.*, 1981); biochemical studies involving comparison of plasma retinol levels in cancer cases and controls (Basu *et al.*, 1976, 1982; Ibrahim *et al.*, 1977; Atukorala *et al.*, 1979; and biochemical studies of retinol status in those who are destined to develop cancer (Wald *et al.*, 1980; Kark *et al.*, 1981). These studies have consistently implied that low plasma or dietary intake of retinol may be associated with increased risk of developing cancer. However, there is little or no information in the literature showing a relationship between retinol status and subsequent cancer recurrence in patients who had undergone curative surgery. The present study was undertaken to investigate whether lower plasma retinol is associated with increased risk of cancer recurrence in post-operative, apparently disease-free colorectal cancer patients.

The mobilization and transportation of retinol from liver storage requires hydrolysis of the retinyl esters followed by conjugation of the free retinol with a specific transport protein, retinol-binding protein (RBP) produced by the liver. The holoprotein is then released to the circulation where it binds to prealbumin as a 1:1 molar complex. The resulting complex transports retinol to target organs (Goodman, 1974; Peterson *et al.*, 1974). In order to determine the metabolically available retinol, the plasma level of RBP was measured in conjunction with retinol.

Correspondence: T.K. Basu.
Received 12 July 1984; and in revised form 9 October 1984.

### Patients and methods

### *Patients*

A 10-year follow-up of 487 cases of colorectal cancer at the W.W. Cross Cancer Institute (WWCCI) in Edmonton, Alberta showed an overall 5-year survival of 35% (McCarten & Preston, 1973). An ongoing randomized prospective surgical adjuvant GI trial involving Dukes' B2 (with full thickness of the bowel wall but no nodal involvement) and C (with regional lymph-node metastases) colorectal carcinoma was initiated in 1976 at the WWCCI. Patients entering into this trial were randomly assigned into one of the three groups including control receiving no adjuvant treatment, immunotherapy, and chemoimmunotherapy. All patients considered for the study were entered as randomized only if informed consent was given. Patients were ineligible if they had had preoperative radiotherapy, chemotherapy or immunotherapy within the previous year. The main assess-

© The Macmillan Press Ltd., 1985

ment of the trial is survival which will be calculated for each patient allocated from the data of definite resection.

The present study was an integral part of the WWCCI trial. A total of 103 patients who were on the control arm of the surgical adjuvant trial were assigned to this investigation. All these patients had previously undergone curative resections of the histologically proven colonic and rectal adenocarcinomas. Sixty-six patients (36 males and 30 females) has a Dukes' B2 tumour, and 37 (18 males and 19 females) had a Dukes' C tumour. The distribution of tumour sites in the patients before surgery is shown in Table I. At the time of diagnosis and surgery, the mean age of the entire group was 62.6 years, with a range between 23 and 80. Over 98% of the patients were over 40 years of age. Sixty-five apparently healthy subjects (34 males and 31 females) who were Red Cross blood donors formed the basis of the controls of the study. Their mean age was 46.5 years with a range between 23 and 65. Over 72% of these subjects were over 40 years. Although such subjects were not an ideal "control" group, their biochemical values provided normal ranges for comparison with the results from the patients, since the same methods were employed in determining the various indices for both the "control" subjects and the post-operative colorectal cancer patients.

**Table I** Distribution of tumour sites in the cancer patients before surgery

| | *Total No.* | |
|---|---|---|
| *Site of Tumour* | *Dukes B2* | *Dukes C* |
| Cecum | 9 | 4 |
| Ascending colon | 11 | 4 |
| Descending colon | 7 | 2 |
| Transverse colon | 3 | 2 |
| Flexures | 6 | 4 |
| Recto-sigmoid | 16 | 16 |
| Rectum | 14 | 5 |
| Totals | 66 | 37 |

*Methods*

Non-fasting blood samples were drawn from all subjects in EDTA tubes. The samples were wrapped with foil during transportation from the WWCCI and the Red Cross Blood Transfusion Clinics to the laboratory, thus minimizing the loss of vitamin A which is sentitive to light. The separated plasma samples were stored at $-35°C$ until analyzed.

Retinol was determined in the plasma by a modification (Steveninck & DeGoeij, 1973) of the fluorometric method of Hansen & Warwick (1968). In this method, fluorescence was measured at an emission wavelength of 550 nm where interference from carotenoids was virtually zero. To determine the metabolically available retinol, plasma concentrations of RBP were determined by the single radial immunodiffusion technique (Mancini *et al.*, 1965) using LC-partigen immunodiffusion plates (Behring Diagnostics).

All biochemical analyses were performed blind, i.e. the classification of the patients was not known during the study. One-way analysis of variance was used to determine the significant differences between the means of the different groups for each of the variables. The student *t*-test was used to determine if there was a significant difference between means of any two groups. A pairwise *t*-test was used to identify any significant change between the first and second samples for patients who had serial samples.

### Results

The plasma concentrations of retinol and RBP in the control subjects did not appear to be subject to age and sex variations (23–65 years). Furthermore, the mean values of the indices obtained in these subjects were found to be similar to those reported by others involving control subjects with age groups matched with the age of the patients (23–80 years) included in the present study (Willett *et al.*, 1984; Stich *et al.*, 1984). The results of our control subjects as a group, irrespective of age and sex, were therefore compared with that of the post-operative colorectal cancer patients. Table II shows the mean differences in plasma retinol and RBP between control subjects and the post-operative colorectal cancer patients who appeared to be disease-free. Both groups (B2 and C) of disease-free cancer patients were found to be associated with not only subnormal plasma retinol levels ($P<0.001$), but also lower RBP levels ($P<0.01$) than the "control" subjects. Statistical difference of the RBP levels between "controls" and the patients

**Table II** Plasma retinol and RBP levels in postoperative colorectal cancer patients

| *Groups* | *Retinol* ($\mu g\,dl^{-1}$) | *RBP* ($mg\,dl^{-1}$) |
|---|---|---|
| Controls | $65.3 \pm 3.2$ | $5.7 \pm 0.3$ |
| Colorectal B2 | $43.5 \pm 1.8^a$ | $5.0 \pm 0.3^b$ |
| Colorectal C | $43.1 \pm 2.9^a$ | $3.8 \pm 0.4^a$ |

Each value is the mean ±s.e.
[a]Significantly different from controls, $P<0.001$.
[b]Significantly different from controls $P<0.01$.

with "C" type colorectal cancer appeared to be more marked ($P<0.001$) than the difference between "controls" and the patients with "B2" type cancer.

No differences between the cancer sites for retinol and RBP were observed as determined by one-way analysis of variance. Patients were grouped together by period intervals from the date of surgery to the date of blood sample collection. The shortest interval (<2 months) was associated with the lowest average mean of plasma retinol and RBP levels, while during the period of 6–12 months following surgery, the average mean values of the indices were found to be at their maximum levels. Analysis of variance, however, revealed no overall significant difference in these parameters between the various intervals of time. Of the 103 patients, 40 had serial samples taken at two different periods of time. The time interval between the first and second samples ranged from less than one month to 4 months with an average time difference of 2–6 months. No significant differences in plasma concentrations of retinol and RBP were observed between the two periods of time. In follow-up studies to date, of the 103 apparently disease-free colorectal cancer patients, a total of 12 subjects (from both B2 and C groups) subsequently had a recurrence of the disease who showed lower plasma levels of retinol as well as RBP, then those who remained disease-free (Table III). However, when the subsequent recurrent cases were removed from the total of 103 patients who were thought to be free from colorectal cancer following surgery, the results of the comparison of the plasma retinol and RBP levels remained the same as those shown in Table II where the recurrent cases were included.

It was interesting to note that plasma concentrations of retinol and RBP were very low in two recurrent patients who died of the disease during this study (Table IV).

**Table III** Plasma retinol and RBP in patients remaining disease-free and in patients with subsequent recurrence following surgery

| *Groups* | *Retinol* ($\mu g\,dl^{-1}$) | *RBP* ($mg\,dl^{-1}$) |
|---|---|---|
| Disease-free (n=91) | $44.5 \pm 1.6$ | $4.6 \pm 0.3$ |
| Recurrence (n=12) | $35.1 \pm 5.2$ | $3.7 \pm 0.4$ |
| Significance (*P* value) | 0.05 | 0.05 |

Each value is the mean ± s.e.

### Discussion

Plasma from healthy blood donors was used merely to standardize the methodologies for both retinol and RBP. Since these subjects had no recent surgery, limited conclusions can be drawn from comparisons of the post-operative colorectal cancer patients' values to these normals. However, it is interesting to note that although the patients were seemingly free of colorectal cancer, they had subnormal levels of plasma retinol when compared with the apparently healthy subjects ($43.3\,\mu g\,dl^{-1}$ *vs* $65.3\,\mu g\,dl^{-1}$). This finding is consistent irrespective of either the stage of the disease or the site of the tumour at the time of diagnosis.

Subnormal levels of plasma retinol have been reported in cancers of the bronchus (Basu *et al.*, 1976); lung (Atukorala *et al.*, 1979); oropharynx (Ibrahim *et al.*, 1977) and GI tract (Abels *et al.*, 1941). In these studies, blood samples were taken from patients who still had a tumour. It is, therefore, possible that their appetite was depressed due to the presence of the tumour, or the tumour itself may have lowered retinol levels due to its increased requirement. Moreover, the treatment of the disease by either chemotherapy or radiotherapy may have resulted in nutritional problems (Dewys & Walters, 1975).

It is noteworthy to point out that the patients in the present study had already undergone resection of their carcinoma, and were believed to be free of neoplastic disease following surgery when the blood samples were collected. In addition, these patients

**Table IV** Plasma retinol and RBP concentrations of the two patients who died during the study

| *Age* (*year*) | *Sex* | *Stage of diagnosis* | *Primary site* | *Survival following surgery* (*months*) | *Retinol* ($\mu g\,dl^{-1}$) | *RBP* ($mg\,dl^{-1}$) |
|---|---|---|---|---|---|---|
| 68 | male | B2 | Sigmoid colon[a] | 5 | 19.32 | 2.40 |
| 78 | male | C | Ascending colon[a] | 8 | 18.86 | 1.68 |

[a]Metastasis to liver.

were not undergoing any kind of therapy, and yet they were found to have subnormal circulating levels of retinol. This was true, not only when the blood samples were collected soon after surgery, but also in the subsequent samples collected 1–4 months later.

The low plasma retinol in the postoperative colorectal cancer patients appears to be associated with subnormal plasma levels of RBP. Since retinol is transported to the circulation bound to RBP, the low plasma retinol in the colorectal cancer patients may be the result of a lower concentration of the carrier protein.

With the exception of one study (Willett *et al.*, 1984), most studies have indicated that subnormal plasma retinol levels exist long before the appearance of a tumour. Thus, low circulating levels of the vitamin were observed in subjects who subsequently developed cancer of epithelial cell origin (Wald *et al.*, 1980; Kark *et al.*, 1981). However, in a case-control study, the plasma concentrations of RBP were found to be normal in samples taken 2–7 years before the development of clinically manifested lung tumours (Haines *et al.*, 1982). While there have been a number of studies showing low concentrations of both plasma retinol and RBP in patients with an established tumour (Atukorala *et al.*, 1979; Basu *et al.*, 1982).

It is possible that the circulating level of RBP could be used as a possible tumour marker. Such a hypothesis is further substantiated by the observations made in the present study where colorectal cancer patients with (Dukes' C) or without (Dukes' B2) regional lymph-node metastases were found to be associated with subnormal plasma retinol levels, while RBP levels appeared to be more markedly affected in patients with regional lymph-node metastases than those without. Furthermore, in follow-up studies to date, besides displaying significantly low plasma retinol values, patients who had a subsequent recurrence of the disease also had lower initial mean RBP values than those who remained free of apparent cancer ($3.7\,mg\,dl^{-1}$ *vs* $4.6\,mg\,dl^{-1}$). Interestingly enough, the lowest initial values of retinol and RBP recorded in the study were seen in the only two patients who had died of the disease at the time of follow-up. However, in view of the fact that there is a considerable overlap in the distribution of RBP levels in the patients who do and those who do not have a recurrence of their tumour, the possible hypothesis that RBP could be a tumour marker needs to be made more cautiously at this stage.

Nonetheless, the results of this study appear to suggest that plasma retinol levels in the post-operative disease-free colorectal cancer patient are lower than those in normals, lower plasma RBP levels may be correlated with more advanced stages of resected disease, and there may be a correlation between poor metabolic vitamin A status and subsequent relapse. These observations may have prognostic and predictive significance in colorectal cancer. Further work is certainly warranted to establish whether retinol supplementations raise the circulatory levels of the vitamin or would this not occur due to metabolic unavailability of retinol as a result of reduced RBP levels.

This project was funded by the Alberta Cancer Board, Research Project No. R-92. We wish to thank Dr. G.B. Hill, Director of the Department of Epidemiology, Alberta Cancer Board, for his advice and guidance in the statistical analyses; Ms. Denise Harbora for her help in reviewing the patients' records; Mr. Jim Perry, Technical Supervisor of the Red Cross Blood Transfusion for supplying the blood samples from the blood donors; and Mr. C. Humphrey and Mr. R. Weingrast, Department of Computing Services for their assistance.

### References

ABELS, J.C., GORHAM, A.T., PACK, G.T. & RHOADS, C.F. (1941). Metabolic studies in patients with cancer of the gastrointestinal tract. I. Plasma vitamin A levels in patients with malignant neoplastic disease, particularly of the gastrointestinal tract. *J. Clin. Invest.*, **20**, 749.

ATUKORALA, S., BASU, T.K., DICKERSON, J.W.T., DONALDSON, D. & SAKULA, A. (1979). Vitamin A, zinc and lung cancer. *Br. J. Cancer*, **40**, 927.

BASU, T.K., DONALDSON, D., JENNER, H., WILLIAMS, D.C. & SAKULA, A. (1976). Plasma vitamin A in patients with bronchial carcinoma. *Br. J. Cancer*, **33**, 119.

BASU, T.K., ROWLANDS, L., JONES, L. & KOHN, J. (1982). Vitamin A and retinol-binding protein in patients with myelomatosis and cancer of epithelial cell origin. *Eur. J. Cancer Clin. Oncol.* **18**, 339.

BJELKE, E. (1975). Dietary vitamin A and human lung cancer. *Int. J. Cancer*, **15**, 561.

DEWYS, W.D. & WALTERS, K. (1975). Abnormalities of taste sensation in cancer patients. *Cancer*, **36**, 1888.

GRAHAM, S., METTLIN, C., MARSHALL, J., PRIORE, R., RZEPKA, T. & SHEDD, D. (1981). Dietary factors in the epidemiology of cancer of the larynx. *Am. J. Epidemiol.*, **113**, 675.

GOODMAN, D.S. (1974). Vitamin A transport and retinol-binding protein metabolism. *Vit. Horm.* **32:** 167.

HAINES, A.P., THOMPSON, S.G., BASU, T.K. & HUNT, R. (1982). Cancer, retinol-binding protein, zinc and copper. Lancet **i**, 52.

HANSEN, L.G. & WARWICK, W.J. (1968). A fluorometric method for the determination of serum vitamin A. *Am. J. Clin. Pathol.*, **50**, 525.

HIRAYAMA, T. (1979). Diet and cancer. *Nutr. Cancer*, **1.**, 67.

IBRAHIM, K., JAFAREY, N.A. & ZEBERI, S.I. (1977). Plasma vitamin A and carotene levels in squamous cell carcinoma of the oral cavity and oro-pharynx. *Clin. Oncol.*, **3**, 203.

KARK, J.D., SMITH, A.H., SWITZA, B.R. & HAMES, C.G. (1981). Serum vitamin A (retinol) and cancer incidence in Evans County, Georgia. *J. Natl Cancer Inst.*, **66**, 7.

MANCINI, G., CARBONARA, A.O. & HEREMAN, J.I. (1965). Immunochemical quantitation of antigens by single radial immunodiffusion. *Immunochemistry*, **2**, 235.

McCARTEN, A.B. & PRESTON, W. (1973). Cancer of the colon and rectum. *Mod. Med. Canada*, **34**, 1458.

METTLIN, C. & GRAHAM, S. (1979). Dietary factors in human bladder cancer. *Am. J. Epidemiol.*, **110**, 235.

PETERSON, P.A., NILSSON, S.F., OSTBERG, L., RASK, L. & VAHLQUIST, A. (1974). Aspects of the metabolism of retinol-binding protein and retinol. *Vit. Horm.*, **32**, 181.

STEVENINCK, J.V. & DEGOEIJ, A.F.P.M. (1973). Determination of vitamin A in blood plasma of patients with carotenaemia. *Clin. Chim. Acta*, **49**, 61.

STICH, H.F., ROSIN, M.P. & VALLEJERA, M.O. (1984). Reduction with vitamin A and beta-carotene administration of proportion of micronucleated buccal mucosal-cells in Asian betel nut and tobacco chewers. *Lancet*, **i**, 1204.

WALD, N., IDLE, M., BOREHAM, J. & BAILEY, A. (1980). Low serum vitamin A and subsequent risk of cancer. *Lancet*, **ii**, 813.

WILLETT, C.W., POLK, B.F., UNDERWOOD, B.A., STAMPFER, M.J., PRESSEL, S., ROSNER, B., TAYLOR, J.O., SCHNEIDER, K. & HAMES, C.G. (1984). Relation of serum vitamins A and E and carotenoids to the risk of cancer. *N. Engl. J. Med.*, **310**, 430.